# Video Laryngoscopy in Emergency Airway Management - a Paradigm Shift from ‘I’ to ‘We’; a Letter to Editor

**DOI:** 10.22037/aaem.v10i1.1474

**Published:** 2022-03-20

**Authors:** Sadaf Sheikh, Faisal Shamim

**Affiliations:** 1 Emergency Physician, Al-Hayat International Hospital, Muscat, Oman.; 2 Intensivist, Aga Khan University Hospital, Karachi, Pakistan.

**Keywords:** Laryngoscopy, airway management, emergencies, teaching, education

## Abstract

Emergency medicine has evolved as a speciality but airway management is still a challenge. Traditionally, direct laryngoscopy (DL) is used for intubation with maneuvers to directly visualize the vocal cords. Most tracheal intubations in the emergency department (ED) are done on an emergent basis and enhancing the technicalities of intubation can be life-saving. Video laryngoscopy (VL) is available in the emrgenyc department and can help reduce the intubation failure rate; hence, it has been recommended for maintaining airways in obese patients.

**Dear Editor;**

Video and optical laryngoscopic devices are valuable changes in the airway management paradigm with the hope of dominating the field of emergent airway management. They contain essential technical details, which are valuable for peri-operative airway assessment. Airway management experts are suggesting the technical benefits of VL. Firstly, it has got a short learning curve for emergency physicians. It is a great tool for operators with no or little experience. It gives a better laryngeal view due to the camera on the blade’s distal end with Macintosh-shaped blades and extra curved blades augment a view that is beyond the reach of Macintosh-styled blades. The force applied while intubating with VL is far less than DL, which helps reduce the trauma to the oral cavity. VL has a higher success rate for intubation when used as the first line or rescue device and keeps the intubation attempts to a minimum (1-3).

Secondly, when the trainer can see the larynx on the screen when a trainee is performing largynoscopy, it helps them give instructions to the trainee to optimize the blade position and the placement of the endotracheal tube (ETT) by marking important landmarks on the screen. Thus, the trainee can complete this procedure without the trainer taking over, which is instrumental in rapid sequence intubations. VL enhances team work and clinical governance as the whole team can see the ETT pass. These merits are even worth considering when intubating critically ill patients in the intensive care units. 

Despite the merits of VL, few challenges are faced during its use and gaining experience. Operators with standard Macintosh laryngoscope cannot equate their skill in handling VL. The same applies to experience with one type of VL and trying to use another type. The reason is that there are numerous VL designs that require various techniques. For instance, C-MAC has been used as a standard Macintosh laryngoscope; whereas, the GlideScope is used without tongue displacement (4, 5). Insertion depth and direction of applied forces require attention as these devices are inserted less far and require the blade to be lifted vertically. This information would help the operator perform better and is vital for patient safety. A reasonable view of vocal cords with VL doesn’t necessarily warrant easy intubation as inexpert handling of VL leads to intubation failure and airway trauma. Some cost-effective options entail ETT with stylets to be flexed while perfoming intubation (5). We suggest using VL with extra-curved blade to enhance chances of seeing round the corner and with a separate screen to make it easier for the trainer to guide a trainee and other members of the team. 

VL is not a great tool when there is a significant limitation of mouth opening not allowing the insertion of the VL blade (5). A difficult scenario could be laryngoscopic paradox, which is defined as a difficult endotracheal passage despite a great view, so one must be aware of such concepts. VL may have had a poorer alignment of oral, pharyngeal and laryngeal axes as there are hyperangulated blades as well as standard geometry blades (5). So we feel the tool is only as good as the operator, so there is a need to train an operator in both DL and VL. 

We studied a combination of elements to address the technical concern of intubation. Knowing the risks and benefits of emergency airway management and devices used is required for safe practice. DL should always be kept as a foremost skill and we advocate the use of VL as a preference skill in patients with cervical spine injuries or restricted mouth opening. It is yet to be determined if VL can cause a paradigm shift from DL in emergent airway management. 

## 1. Declarations:

### 1.1. Acknowlegment:

None.

### 1.2. Authors’Contributions:

SS conceived the manuscript idea and drafting and FS supervised the manuscript formation**. **

### 1.3. Conflict of interest:

None to declare.

### 1.4. Funding and supports:

None to declare.
